# Supplementary material for: Extracting medicinal chemistry intuition via preference machine learning
Source: Nat Commun. 2023 Oct 31;14:6651. doi: 10.1038/s41467-023-42242-1 (PMC10618272; doi:10.1038/s41467-023-42242-1)
Supplement: Supplementary file 1 — Supplementary Information [file 41467_2023_42242_MOESM1_ESM.pdf]

Extracting medicinal chemistry intuition via preference machine learning

Supporting Information

Oh-Hyeon Choung[1], Riccardo Vianello[1], Marwin Segler[2], Nikolaus Stiefl[1,*], and José Jiménez-Luna[2,*]

[1]Novartis Institutes for Biomedical Research, 4002 Basel, Switzerland
[2]Microsoft Research AI4Science, CB1 2FB Cambridge, United Kingdom
[*]Correspondence: nikolaus.stiefl@novartis.com, jjimenezluna@microsoft.com

List of Figures

S1 **Preliminary-round inter-rater agreements**. Two-by-two inter-rater agreement coefficients between the chemists for the first (above), and second (below) preliminary round of the study. Inter-rater agreements measured with both Cohen's κ_C coefficient and accuracy metrics. . . . 2

S2 **Model predictive performance as a function of different combinations of molecular descriptors**. Performance, as measured by the area under the receiver operating characteristic curve (AUROC) and tested on both the collected preliminary data (n = 440) and via 5-fold cross-validation (average performance over the folds, ±1 standard deviation) at different training set sizes during the production run. 3

S3 **Relationship between learned scores and other cheminformatics metrics**. Average absolute correlation coefficients (±1 standard deviation) between several *in silico* descriptors computed via RDKit and learned compound scores (lower is better) in the training set (n = 5276). 4

S4 **Discriminative performance between chemical sets as a function of molecular size**. Performance dependency, as measured by ROC-AUC, of both MolSkill and QED scores on the number of heavy atoms when distinguishing molecules from a 'drug-like' set (*i.e.*, ChEMBL31, FDA-approved drugs from DrugBank, 2386 and 732 compounds, respectively) from a combinatorially-generated one (*i.e.*, the GDB13-17 sets, 8616 compounds). Results obtained by filtering the drug-like sets to feature at most k heavy atoms in each comparison. . 9

S5 **Comparing QED and Molskill scores as a function of molecular size.** Distribution of QED and MolSkill values versus the number of heavy atoms for all the molecules extracted from the ChEMBL (a) and the FDA-approved drugs (b) sets used throughout this study. . . . 10

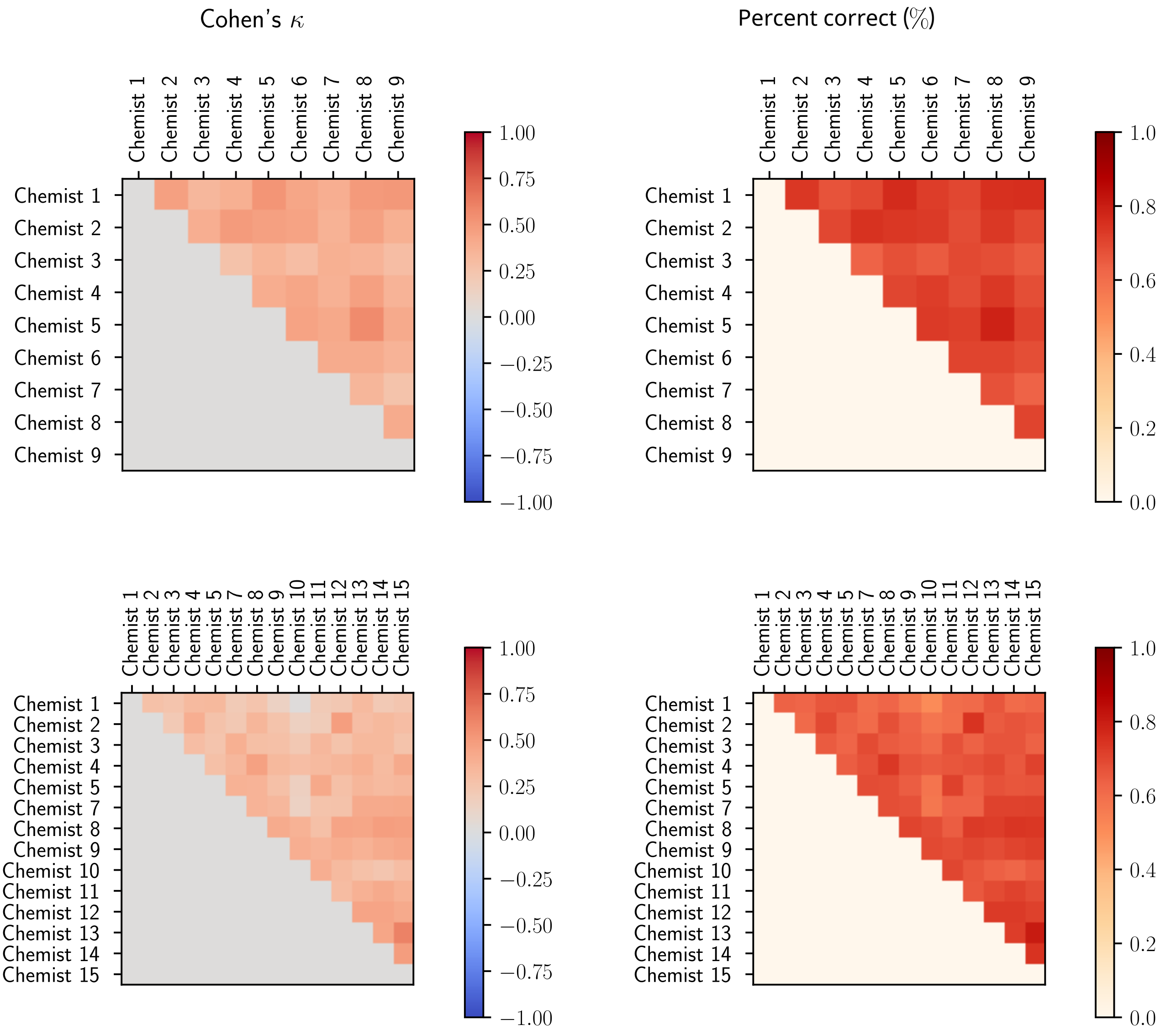

Figure S1: Preliminary-round inter-rater agreements. Two-by-two inter-rater agreement coefficients between the chemists for the first (above), and second (below) preliminary round of the study. Inter-rater agreements measured with both Cohen's κ_C coefficient and accuracy metrics.

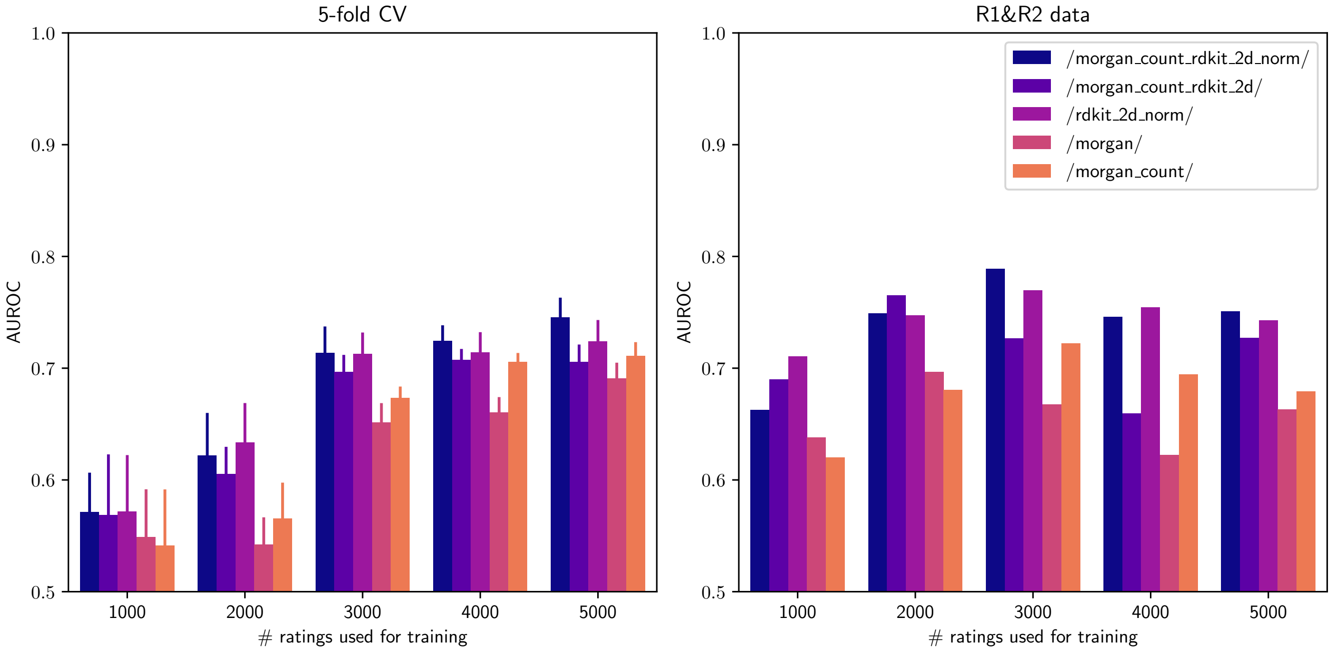

Figure S2: Model predictive performance as a function of different combinations of molecular descriptors. Performance, as measured by the area under the receiver operating characteristic curve (AUROC) and tested on both the collected preliminary data ($n = 440$) and via 5-fold cross-validation (average performance over the folds, ± 1 standard deviation) at different training set sizes during the production run.

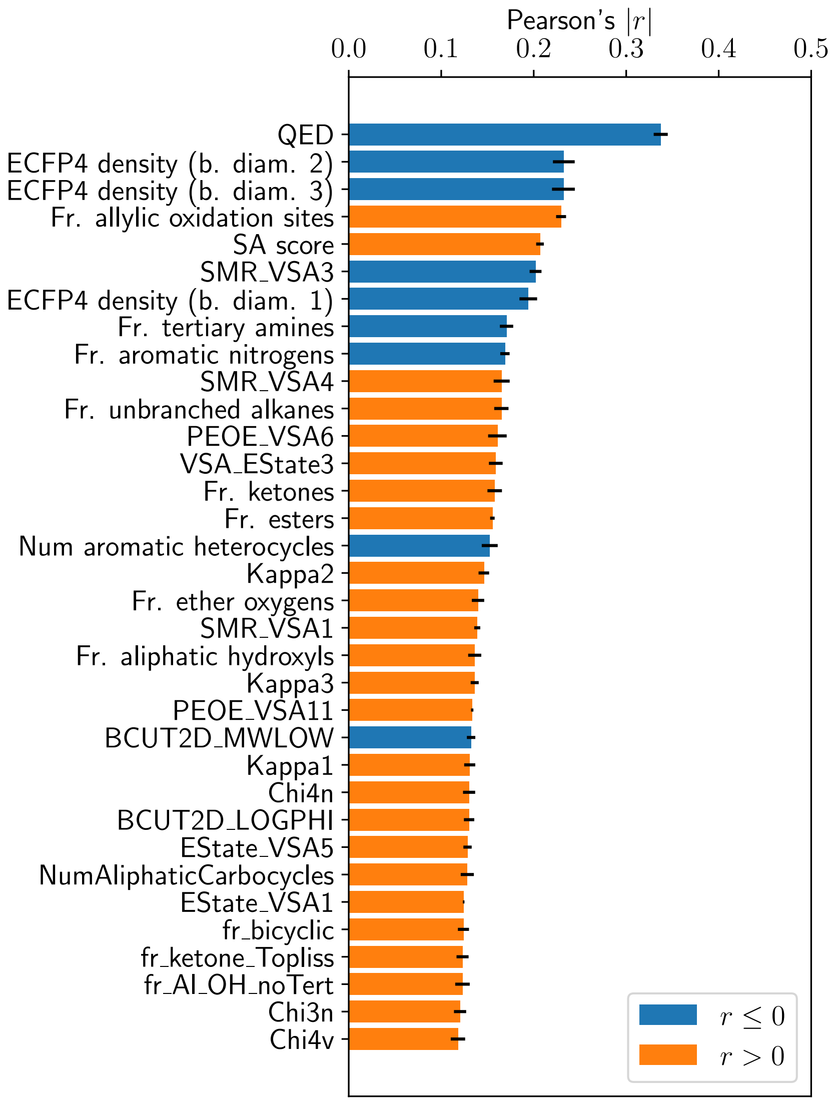

Figure S3: Relationship between learned scores and other cheminformatics metrics. Average absolute correlation coefficients (± 1 standard deviation) between several *in silico* descriptors computed via RDKit and learned compound scores (lower is better) in the training set ($n = 5276$).

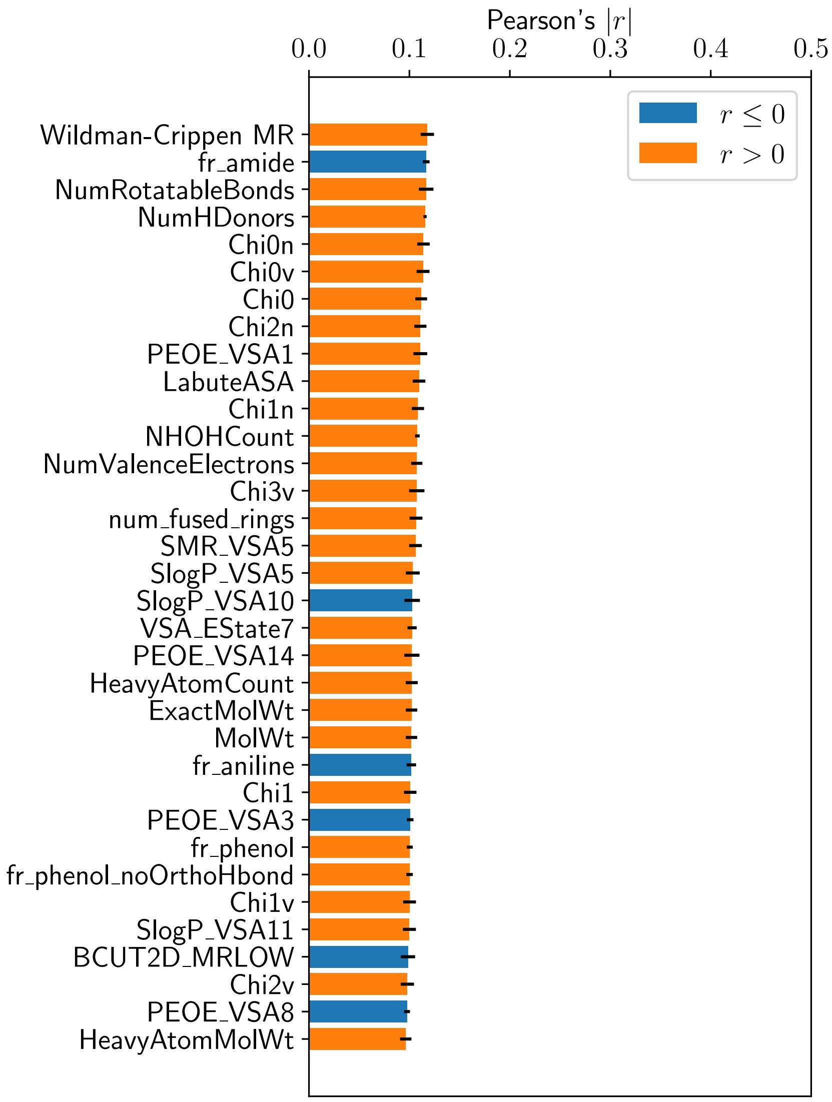

Figure S3: (continued) **Relationship between learned scores and other cheminformatics metrics**. Average absolute correlation coefficients (±1 standard deviation) between several *in silico* descriptors computed via RDKit and learned compound scores (lower is better) in the training set ($n = 5276$).

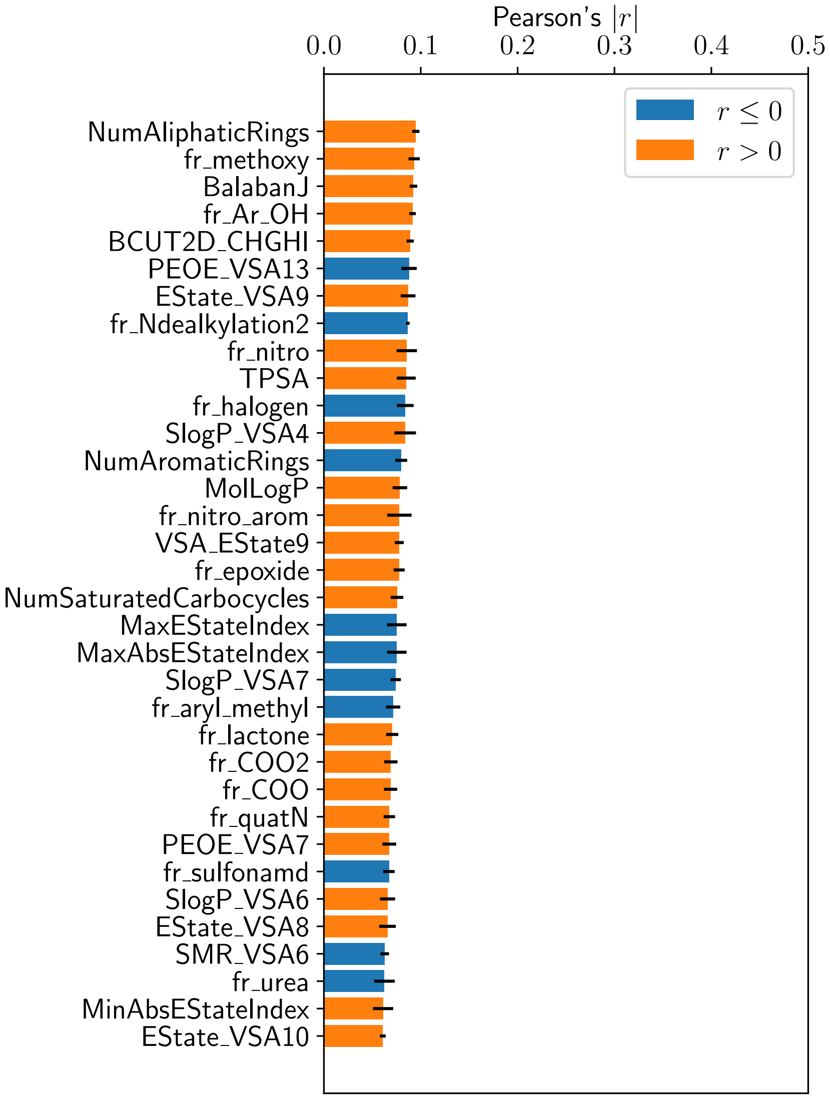

Figure S3: (continued) **Relationship between learned scores and other cheminformatics metrics**. Average absolute correlation coefficients (±1 standard deviation) between several *in silico* descriptors computed via RDKit and learned compound scores (lower is better) in the training set ($n = 5276$).

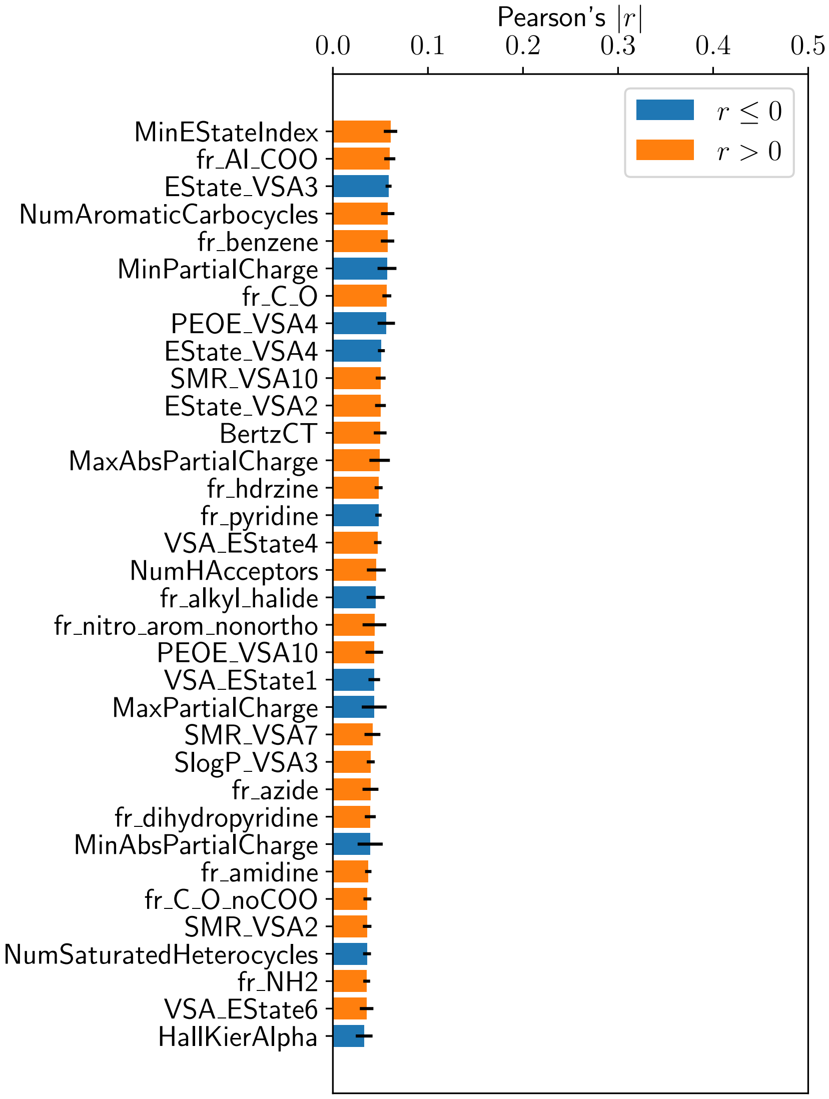

Figure S3: (continued) **Relationship between learned scores and other cheminformatics metrics**. Average absolute correlation coefficients (±1 standard deviation) between several *in silico* descriptors computed via RDKit and learned compound scores (lower is better) in the training set ($n = 5276$).

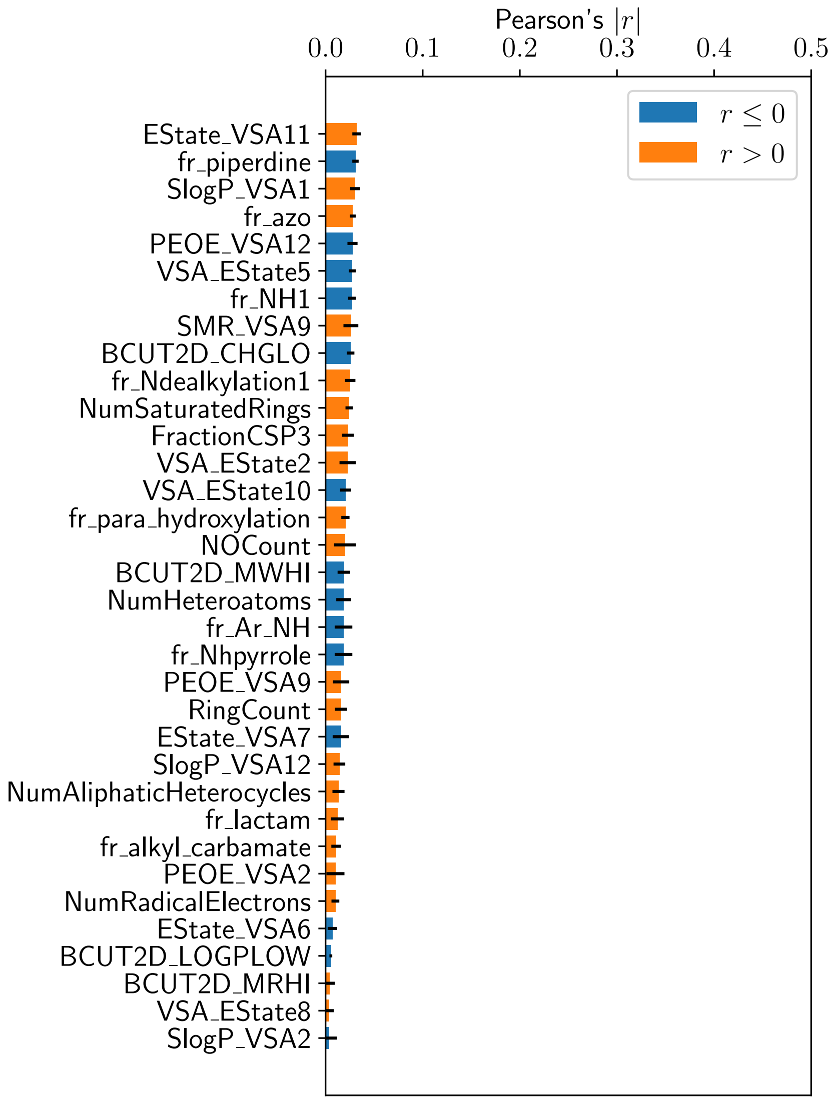

Figure S3: (continued) **Relationship between learned scores and other cheminformatics metrics**. Average absolute correlation coefficients (± 1 standard deviation) between several *in silico* descriptors computed via RDKit and learned compound scores (lower is better) in the training set ($n = 5276$).

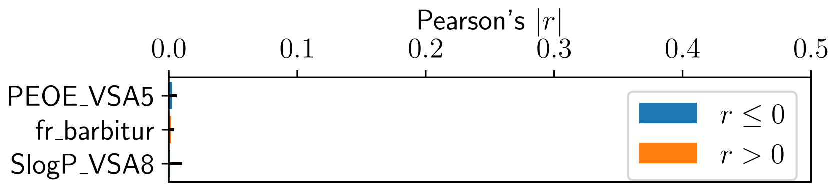

Figure S3: (continued) **Relationship between learned scores and other cheminformatics metrics**. Average absolute correlation coefficients (± 1 standard deviation) between several *in silico* descriptors computed via RDKit and learned compound scores (lower is better) in the training set ($n = 5276$).

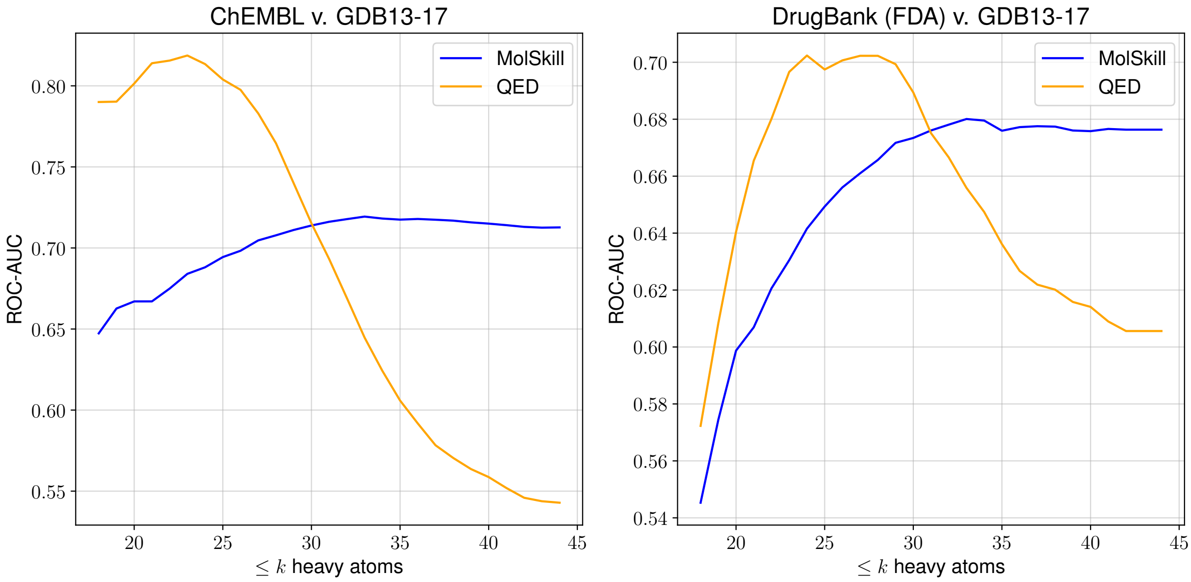

Figure S4: Discriminative performance between chemical sets as a function of molecular size. Performance dependency, as measured by ROC-AUC, of both MolSkill and QED scores on the number of heavy atoms when distinguishing molecules from a 'drug-like' set (*i.e.*, ChEMBL31, FDA-approved drugs from DrugBank, 2386 and 732 compounds, respectively) from a combinatorially-generated one (*i.e.*, the GDB13-17 sets, 8616 compounds). Results obtained by filtering the drug-like sets to feature at most k heavy atoms in each comparison.

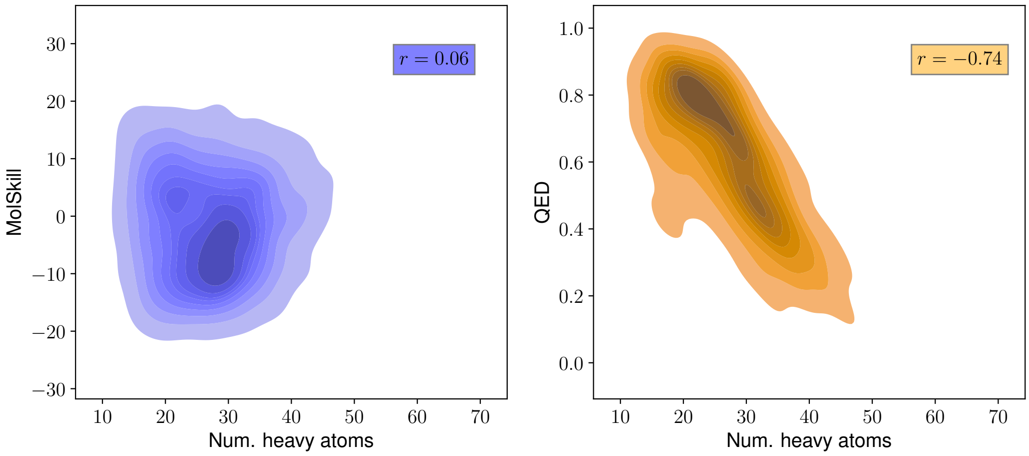

(a)

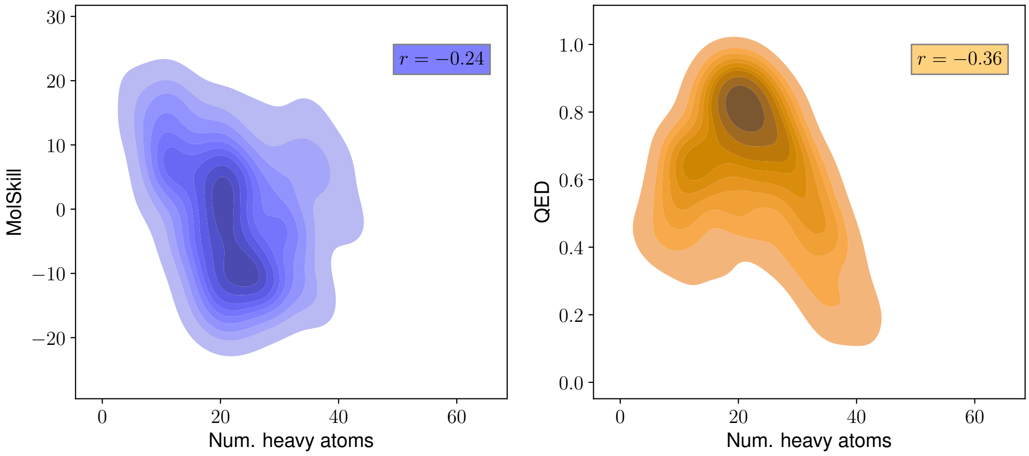

(b)

Figure S5: Comparing QED and Molskill scores as a function of molecular size. Distribution of QED and MolSkill values versus the number of heavy atoms for all the molecules extracted from the ChEMBL (a) and the FDA-approved drugs (b) sets used throughout this study.
