# Supplementary material for: Extracting medicinal chemistry intuition via preference machine learning
Source: Nat Commun. 2023 Oct 31;14:6651. doi: 10.1038/s41467-023-42242-1 (PMC10618272; doi:10.1038/s41467-023-42242-1)
Supplement: Supplementary file 3 — Reporting Summary [file 41467_2023_42242_MOESM3_ESM.pdf]

nature portfolio

Corresponding author(s): NCOMMS-23-10135-T

Last updated by author(s): Aug 11, 2023

Reporting Summary

Nature Portfolio wishes to improve the reproducibility of the work that we publish. This form provides structure for consistency and transparency in reporting. For further information on Nature Portfolio policies, see our Editorial Policies and the Editorial Policy Checklist.

Statistics

For all statistical analyses, confirm that the following items are present in the figure legend, table legend, main text, or Methods section.

n/a	Confirmed	
☒	☐	The exact sample size (n) for each experimental group/condition, given as a discrete number and unit of measurement
☒	☐	A statement on whether measurements were taken from distinct samples or whether the same sample was measured repeatedly
☒	☐	The statistical test(s) used AND whether they are one- or two-sided<br>*Only common tests should be described solely by name; describe more complex techniques in the Methods section.*
☒	☐	A description of all covariates tested
☒	☐	A description of any assumptions or corrections, such as tests of normality and adjustment for multiple comparisons
☒	☐	A full description of the statistical parameters including central tendency (e.g. means) or other basic estimates (e.g. regression coefficient) AND variation (e.g. standard deviation) or associated estimates of uncertainty (e.g. confidence intervals)
☒	☐	For null hypothesis testing, the test statistic (e.g. F, t, r) with confidence intervals, effect sizes, degrees of freedom and P value noted<br>*Give P values as exact values whenever suitable.*
☒	☐	For Bayesian analysis, information on the choice of priors and Markov chain Monte Carlo settings
☒	☐	For hierarchical and complex designs, identification of the appropriate level for tests and full reporting of outcomes
☒	☐	Estimates of effect sizes (e.g. Cohen's d, Pearson's r), indicating how they were calculated

Our web collection on statistics for biologists contains articles on many of the points above.

Software and code

Policy information about availability of computer code

Data collection	all code is open source and available on github https://github.com/microsoft/molskill
Data analysis	all code is open source and available on github https://github.com/microsoft/molskill

For manuscripts utilizing custom algorithms or software that are central to the research but not yet described in published literature, software must be made available to editors and reviewers. We strongly encourage code deposition in a community repository (e.g. GitHub). See the Nature Portfolio guidelines for submitting code & software for further information.

Data

Policy information about availability of data

All manuscripts must include a data availability statement. This statement should provide the following information, where applicable:

- Accession codes, unique identifiers, or web links for publicly available datasets
- A description of any restrictions on data availability
- For clinical datasets or third party data, please ensure that the statement adheres to our policy

Data is available on github https://github.com/microsoft/molskill

Human research participants

Policy information about studies involving human research participants and Sex and Gender in Research.

Reporting on sex and gender	No explicit information on sex/gender has been collected in this study.<br>Additionally, only consent for the sharing of anonymized group-level study data was provided.
Population characteristics	All chemists recruited were working at Novartis at the time of the study.<br>9 and 14 chemists were recruited for the first and second preliminary rounds, while 35 were recruited for the production rounds of the study.
Recruitment	Chemists were internally recruited at Novartis via email at different geographical sites (CH/US), at different levels of seniority, and from different backgrounds: medicinal, organic, analytical or computational.
Ethics oversight	Novartis / Microsoft Research

Note that full information on the approval of the study protocol must also be provided in the manuscript.

Field-specific reporting

Please select the one below that is the best fit for your research. If you are not sure, read the appropriate sections before making your selection.

☐ Life sciences ☒ Behavioural & social sciences ☐ Ecological, evolutionary & environmental sciences

For a reference copy of the document with all sections, see nature.com/documents/nr-reporting-summary-flat.pdf

Behavioural & social sciences study design

All studies must disclose on these points even when the disclosure is negative.

Study description	The study collected quantitative data in the form of human preferences between pairs of molecular compounds
Research sample	9 and 14 chemists at Novartis were recruited for the first and second preliminary rounds, while 35 were recruited for the production rounds of the study.
Sampling strategy	The selection of chemists inside Novartis was not randomized, with the goal of obtaining as many participants as possible in the study.
Data collection	Data was collected via a web application that could be accessed via a computer or a touchscreen device.
Timing	Preliminary study data collected June-Sept 2022. Production round data collected between Sept. 2022 and Jan. 2023
Data exclusions	No explicit data exclusions to report.
Non-participation	All participants opted-in the study. Only one chemist dropped out between preliminary rounds 1 and 2 for time reasons.
Randomization	Participants were not allocated into experimental groups.

Reporting for specific materials, systems and methods

We require information from authors about some types of materials, experimental systems and methods used in many studies. Here, indicate whether each material, system or method listed is relevant to your study. If you are not sure if a list item applies to your research, read the appropriate section before selecting a response.

Materials & experimental systems

n/a	Involved in the study
☒	☐ Antibodies
☒	☐ Eukaryotic cell lines
☒	☐ Palaeontology and archaeology
☒	☐ Animals and other organisms
☒	☐ Clinical data
☒	☐ Dual use research of concern

Methods

n/a	Involved in the study
☒	☐ ChIP-seq
☒	☐ Flow cytometry
☒	☐ MRI-based neuroimaging
